# Juvenile idiopathic arthritis with dry synovitis: clinical and imaging aspects in a cohort of 6 patients

**DOI:** 10.1186/1546-0096-9-S1-P173

**Published:** 2011-09-14

**Authors:** L De Somer, K Lambot, C Wouters, B Bader-Meunier

**Affiliations:** 1 Department of Pediatric Rheumatology, University Hospitals Leuven, Leuven, Belgium; 2 Service de Radiologie Pédiatrique, Hôpital Necker Enfants Malades, Paris, France; 3 Service d’Immunologie, hématologie et rhumatologie pediatrique, Hôpital Necker Enfants Malades, Paris, France

## Background

Dry synovitis is a rare form of juvenile idiopathic arthritis, incompletely understood and often following a destructive course. Few reports mention this entity within the spectrum of JIA [1,2].

## Aim

To describe the clinical and radiological manifestations and course of dry synovitis.

## Methods

A retrospective study of 6 patients in 2 pediatric rheumatology centers who presented with 1. progressive articular limitations without palpable synovitis and 2. radiologic signs of articular damage. Neuromuscular conditions were excluded. Clinical, laboratory and imaging (X-rays, MRI) data were reviewed.

## Results

The cohort included 2 boys and 4 girls, median (range) age was 9.7yr(3-15). The median (range) age at first manifestation and diagnosis were respectively 4.5yr(1.3-8.9) and 8.3yr(3-14). Presenting signs comprised delayed motor development and/or progressive articular stiffness. Little or no pain was mentioned. Clinical examination showed a symmetric and polyarticular involvement without obvious clinical signs of synovitis. CRP was normal and ESR<35mm/h. In 5/6 patients ANA was absent. Radiological imaging (wrist and/or pelvis) comprised osteopenia, advanced bone age, irregular bone contours and/or erosions in all patients. MRI revealed synovial thickening in 5/6 patients; bone edema was variably present and bone erosions were demonstrated in 4/6 patients. Treatments comprised NSAIDs(n=5/6), corticosteroids(n=3/6), methotrexate(n=5/6), anti-TNF(n=5/6) and resulted in subjective improvement. However articular limitations persisted and imaging showed progressive articular damage.

## Conclusion

Our study confirms progressive, symmetric polyarticular limitations to be the main feature of dry JIA, often causing diagnostic delay. MRI reveals synovial inflammation and demonstrates destructive changes early in the disease course. The exact nature of this entity and a possible contribution of a metabolic or intrinsic bone disorder into its pathogenesis remain to be determined.
